# Losses and External Outcomes Interact to Produce the Gambler’s Fallacy

**DOI:** 10.1371/journal.pone.0170057

**Published:** 2017-01-26

**Authors:** Julia A. Mossbridge, Christopher J. R. Roney, Satoru Suzuki

**Affiliations:** 1 Department of Psychology Northwestern University Evanston Illinois, United States of America; 2 Department of Psychology King’s University College at the University of Western Ontario London, Ont. Canada; 3 Interdepartmental Neuroscience Program Northwestern University Evanston Illinois, United States of America; Universiteit Gent, BELGIUM

## Abstract

When making serial predictions in a binary decision task, there is a clear tendency to assume that after a series of the same external outcome (e.g., heads in a coin flip), the next outcome will be the opposing one (e.g., tails), even when the outcomes are independent of one another. This so-called “gambler’s fallacy” has been replicated robustly. However, what drives gambler’s fallacy behavior is unclear. Here we demonstrate that a run of the same external outcome by itself does not lead to gambler’s fallacy behavior. However, when a run of external outcomes is accompanied by a concurrent run of failed guesses, gambler’s fallacy behavior is predominant. These results do not depend on how participants’ attention is directed. Thus, it appears that gambler’s fallacy behavior is driven by a combination of an external series of events and a concurrent series of failure experiences.

## Introduction

People often try to predict the outcomes of future events regardless of whether the outcomes are possible to predict. This tendency may be adaptive in many or most situations, where there is a predictable pattern. It seems to be problematic, however, when the tendency persists even when outcomes are random, as in online or casino gambling. Over the last half century, trying to understand what outcomes people will predict in a given situation has become a subfield of both behavioral economics and neuroscience [1, 2]. A subset of this work has focused on predictions that people make after a series of binary outcomes or events (for a review, see [3]). In random sequences it is difficult to predict an individual’s bet on the next event unless that individual has experienced a run of consistent external outcomes—like a series of “heads” in a coin toss. After such a run, it is clear that most individuals predict a change in the outcome of the next event (e.g., “tails”). This so-called “gambler’s fallacy,” the belief that external outcomes will reverse after a run even when each outcome is independent and occurs at a fixed probability, has been observed for more than two centuries [4]. Here we present a series of experiments demonstrating that it is the interplay between runs of external outcomes and prediction outcomes, rather than runs of external outcomes per se, that influences people’s future predictions of random outcomes.

To our knowledge there is only one study that has examined this question in relationship to gambler’s fallacy responding [5]. Boynton reported that during a run of external outcomes (e.g., a series of “tails” in a coin toss) people were more likely to respond according to the gambler’s fallacy (e.g., predicting “heads”) on a given trial after having predicted a wrong outcome (e.g., “heads”) on the preceding trial. Nevertheless, Boynton did not systematically manipulate runs of external and prediction outcomes to determine whether gambler’s fallacy responses depended on the concurrent of runs of external outcomes and failed prediction outcomes. Ayton and Fischer [6] demonstrated that runs of prediction outcomes (i.e., success versus failure) affected people’s confidence in their choices, but they did not examine how this influence on confidence might interact with external outcomes to influence people’s predictions. As people appear to be sensitive to runs of both types, in the present study we systematically manipulated external outcomes and prediction outcomes to provide an integrative understanding of how these runs interactively affect predictions of subsequent events.

To this end, we presented participants with a series of guessing tasks based on outcomes of a presumably random system—rolls of a pair of dice summing to an even or odd number—with the outcomes clandestinely controlled to either ensure a run of external outcomes (dice sums) or prediction outcomes (failures or successes). Because it is possible that if participants’ attention is focused on prediction successes and failures, runs of these prediction outcomes could have more influence on gambler’s fallacy responding than when attention is focused on runs of external outcomes, we also manipulated participants’ attentional focus by guiding their attention towards the type of run that was being controlled.

We systematically examined the potential interactive effects of runs of external outcomes and prediction outcomes on how people predicted the next external outcome by comparing responses across the following situations: (1) after experiencing a run of external outcomes alone (without a concurrent run of prediction outcomes), (2) after experiencing a run of external outcomes with a concurrent run of successful prediction outcomes, (3) after experiencing a run of external outcomes with a concurrent run of failed prediction outcomes, (4) after experiencing a run of successful prediction outcomes alone, without a concurrent run of external outcomes, and (5) after experiencing a run of failed prediction outcomes alone, without a concurrent run of external outcomes. These situations were generated using four experimental conditions (see below) to determine which of these situations maximized gambler’s fallacy behavior. Finally, we examined the generality of our results by re-analyzing previously published data obtained from an experiment using a different paradigm.

## Experiment 1 Materials and Methods

### Participants

One hundred participants (45 female; ages 18–70) gave online informed consent to participate in the study. After reading the consent information, participants clicked on a button indicating that they had read and consented to the protocol. Written consent could not be obtained because the experiment was online. Participants were not allowed to continue in the experiment unless they consented in this way. We documented participant consent via a database that recorded button presses. This consent method and all procedures in the first three experiments in this study were approved by the Northwestern University Institutional Review Board. Participants were enrolled via an ad placed on Amazon Mechanical Turk that invited them to participate in an experiment in which they would be asked to determine the values on a set of pictures of dice, and make imaginary bets about the values on future pictures of dice. Previous studies have suggested that data obtained from participants recruited this way are similar to those obtained in a standard psychology laboratory (e.g. [7,8]).

### Experimental conditions

Each of four experimental conditions consisted of a series of 22 trials. On each trial, participants were asked to predict whether the sum of a pair of dice shown on the next trial would be odd or even. They were also asked to rate their confidence in their prediction. The confidence rating was on a scale similar to those used in previous work [9, 10]. Because confidence ratings were not reliably influenced by any of the experimental manipulations, they are not discussed further.

Prior to the experiment, participants were given the following instructions:

You will be asked to do three things when you see each image of a pair of dice: 1) determine whether the SUM of the numbers on their faces is odd or even (not the individual die faces, but the SUM of the die faces), 2) make a guess about whether the next picture of dice will have an odd or even SUM, and 3) rate your confidence in your guess from 0–100%. 100% would mean complete confidence, 50% would mean moderate confidence in your guess, and 0% would indicate a complete lack of confidence.

NOTE: To rate your confidence as 0%, you need to choose another value, then move the pointer back to 0%, or else the software will think you haven't responded to the question.

AS YOU DO THIS LAST STEP, PLEASE IMAGINE YOU ARE BETTING BASED ON YOUR CONFIDENCE. IN OTHER WORDS, YOU COULD IMAGINE THAT INSTEAD OF PERCENTAGE POINTS, WE ARE TALKING ABOUT $0-$100 OF FAKE MONEY.

To control for any sequential effects other than the manipulated aspects, we created a controlled single sequence of trials that was used for all conditions and all participants. The sequence was: AABABBABAABAAAA**B**ABBABB (each letter indicating a trial event). The bolded and underlined trial in this sequence was the critical trial, as it followed a run of four events of the same type. For all conditions, the prediction made for this critical trial was the dependent variable. When runs of an external (dice) outcome were manipulated in the controlled external-outcome conditions “A” and “B” in this sequence represented even and odd sums on the dice or vice versa. When runs of a prediction outcome were manipulated in the controlled prediction-outcome conditions, “A” and “B” represented correct and incorrect predictions or vice versa.

#### Controlled external-outcome (odd vs. even) conditions

**Fig 1:**
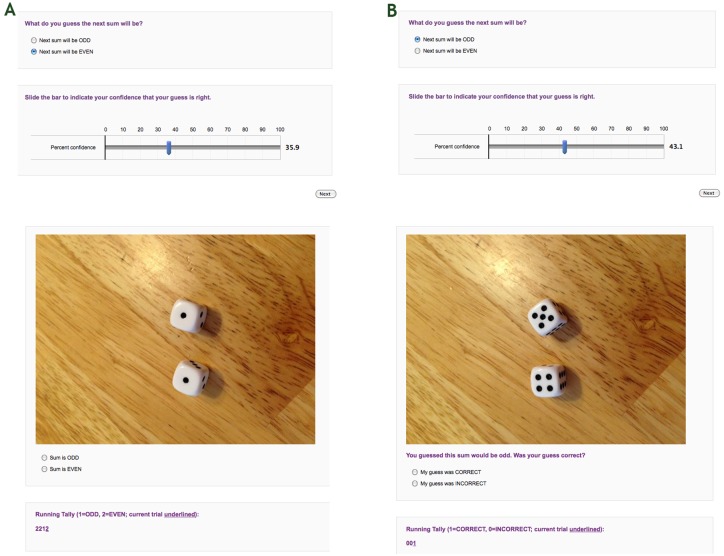
Example trials for each condition type. Example trials from one of the controlled external-outcome conditions (A) and one of the controlled prediction-outcome conditions (B). The top panels show the prediction screens, and the bottom panels show the outcome screens.

Participants performed two controlled external-outcome conditions, called the even- and odd-run conditions (see below and Fig 1A). In these conditions, participants experienced a run of four odd outcomes in the odd-run condition or four even outcomes in the even-run condition. After each picture of dice was shown, participants were required to check a radio button next to “Even” or “Odd” in response to the sum on the dice. To further accentuate the run of external outcomes, a running tally of the sequence of even and odd outcomes was also shown beneath these radio buttons.

1. Even-Run Condition. In this condition, “A” in the sequence AABABBABAABAAAA**B**ABBABB represented a picture of dice with an even sum, while B represented a picture of dice with an odd sum. Thus, the critical trial occurred following a run of “even” outcomes.
2. Odd-Run Condition. In this condition, “A” in the sequence AABABBABAABAAAA**B**ABBABB represented a picture of dice with an odd sum, while B represented a picture of dice with an even sum. Thus, the critical trial occurred following a run of “odd” outcomes.

#### Controlled prediction-outcome (correct vs. incorrect) conditions

Participants performed two controlled prediction-outcome conditions, called the correct-run and incorrect-run conditions (see below and Fig 1B). In these conditions, participants experienced a run of four correct outcomes in the correct-run condition, or four incorrect outcomes in the incorrect-run condition. After each picture of dice was shown, participants were required to check a radio button next to “Correct” or “Incorrect” in response to the match or lack thereof between their prediction for this trial and the outcome shown. As a reminder, their prediction for this trial was also displayed. To further accentuate the run of prediction outcomes, a running tally of the sequence of incorrect and correct predictions was shown beneath the radio buttons.

1. Correct-run condition. In this condition, “A” in the sequence AABABBABAABAAAA**B**ABBABB represented a picture of dice with a sum that matched the previous prediction, while B represented a picture of dice with a sum that did not match the previous prediction. Thus, in this condition the critical trial occurred after a run of four correct predictions.
2. Incorrect-run condition. In this condition, “A” in the sequence AABABBABAABAAAA**B**ABBABB represented a picture of dice with a sum that did not match the previous prediction, while B represented a picture of dice with a sum that did match the previous prediction. Thus, in this condition the critical trial occurred after a run of four incorrect predictions.

### Procedure

All participants performed all four conditions, which were presented online via Qualtrics, a survey-coding software that presented stimuli and recorded self-paced responses. The software ensured that participants answered each question and made a prediction before proceeding to the next trial. Condition type was blocked and the order was counterbalanced across participants. After completion of all four blocks, participants reported demographic information including gender, age, gambling frequency in the past year, nationality, and native language. These demographic factors did not produce significant effects, and are not discussed further.

### Data validation

We aimed to eliminate data from participants who showed evidence of responding without attending to the stimuli or the task. We used two methods to reach this aim. First, data from any participant who responded incorrectly to the questions “Was your guess correct or incorrect” (controlled prediction-outcome conditions) or “Is the sum odd or even?” (controlled external-outcome conditions) on any of the four trials preceding the critical trial were eliminated from the analysis. We removed these data because we could not be sure that these individuals correctly perceived the run, and therefore it was not clear whether their response reflected the actual stimuli or their potentially incorrect belief as to what occurred. This procedure eliminated data from 13 participants. Second, we eliminated from the analysis data from any participant whose guesses on every trial up to the first trial of the run had a standard deviation of zero in any condition. We eliminated data from these individuals because we suspected they might be habitually pressing the same button in an attempt to speed through the task rather than trying to predict the next sum of the dice. This procedure eliminated 15 participants. The analyses presented in the *Results* section include data from the 72 participants remaining after removing data from these 28 participants who apparently were not attending to the task.

### Data analysis

As described in the Introduction, previous work suggests that external outcomes and prediction outcomes may work in combination [5]. To further test this idea, we examined the joint effects of dice-outcome and prediction-outcome runs by comparing results across participants. For the controlled external-outcome conditions, in which all participants received a run of even or odd dice outcomes, we compared the results from (1) the participants who experienced a dice-outcome run along with a concurrent correct-prediction-outcome run, (2) the participants who experienced a dice-outcome run along with a concurrent incorrect-prediction-outcome run, and (3) the participants who experienced a dice-outcome run without a concurrent run of correct or incorrect prediction outcomes. For the controlled prediction-outcome conditions, in which all participants received a run of correct or incorrect prediction outcomes, we compared the results from (4) the participants who experienced a prediction-outcome run along with a concurrent dice-outcome run (even or odd) and (5) the participants who experienced prediction-outcome run without a concurrent dice-outcome run. To obtain adequate sample sizes for all these categories, we considered a run to consist of two or more repetitions of dice and/or prediction outcomes immediately preceding the critical trial. We examined how the probability of choosing a reversal in the dice outcome (i.e., the probability of gambler’s fallacy responding) was jointly or separately influenced by concurrent runs of dice and prediction outcomes.

Because participants may have baseline tendencies to predict a changed-dice outcome that may deviate from 50%, we compared the proportion of a changed-dice outcome for the critical trial with the baseline tendency to predict a changed-dice outcome for each participant. The baseline tendency was obtained by calculating the proportion of changed-dice outcome predictions on the first 12 trials preceding the run (**A-A-*****B*****-A-B-B-*****A*****-B-A-A-*****B*****-A**-A-A-A-B-A-B-B-A-B-B [bolded trials]) excluding predictions given after repeated dice outcomes (italicized and underlined trials). We note that these comparisons against the individual participants’ baseline tendencies to predict a changed-dice outcome (using Wilcoxon signed-rank test; W and r calculated as in [11]) yielded results that are statistically indistinguishable from comparisons against the chance level of 50% (using Binomial test). This indicates that participants were overall unbiased for or against predicting a changed-dice outcome in the absence of a run of a given dice outcome. We thus present the effect sizes using Binomial z-scores in Table 1.

**Table 1:** Experiments 1–3: Summary statistics. *z*-scores for binomial tests versus chance (50%) the number of participants predicting a changed dice outcome (i.e., gambler’s fallacy) versus continuation of the dice outcome on the post-run critical trial. Positive values indicate that more participants predicted a change in the dice outcome (e.g., predicting an even sum after an odd sum), while negative values indicate that more participants predicted that the previous dice outcome would continue (e.g., predicting an even sum after an even sum). Asterisks indicate significance at *p*<0.001. Where asterisks do not appear, the binomial test was not significant at *p*<0.05.

	Condition (run type)	Dice Only	Correct Only	Incorrect Only	Dice + Correct	Dice + Incorrect
**Experiment 1**	Even	**-0.2** (N = 25)	--	--	**-1.5** (N = 11)	4.67*** (N = 36)
	Odd	**1.0** (N = 25)	--	--	**0.00** (N = 6)	5.15*** (N = 41)
	Correct	--	**1.91** (N = 33)	--	**1.12** (N = 39)	--
	Incorrect	--	--	**-1.53** (N = 21)	--	3.50*** (N = 51)
**Experiment 2**	Correct	--	**1.85** (N = 42)	--	**0.16** (N = 41)	--
	Incorrect	--	--	**-1.77** (N = 32)	--	3.78*** (N = 51)
**Experiment 3**	Even	**0.00** (N = 28)	--	--	**-0.83** (N = 13)	5.81*** (N = 45)
	Odd	**0.19** (N = 29)	--	--	**-1.00** (N = 9)	4.72*** (N = 46)

## Experiment 1 Results

**Fig 2:**
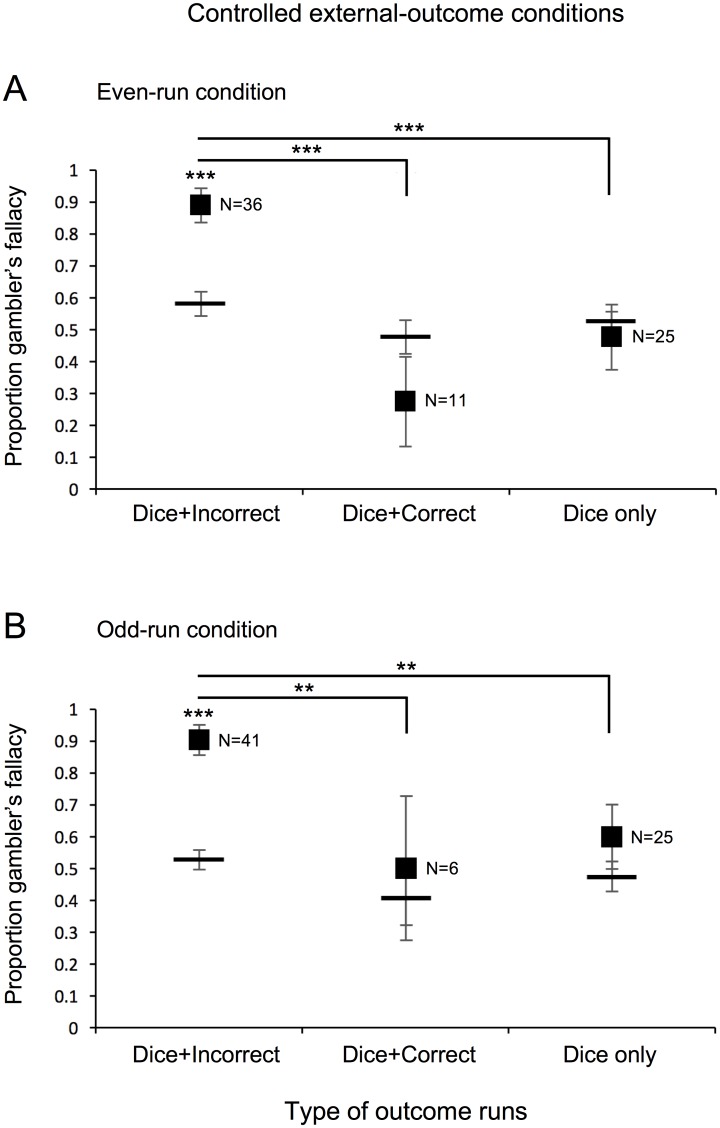
Experiment 1: Controlled external-outcome conditions. Proportion of participants predicting a changed-dice outcome (gambler’s fallacy) on the critical trial in the controlled external-outcome conditions following a run of (A) even or (B) odd dice outcomes. Participants are grouped according to whether they concurrently had a run of incorrect predictions (Dice+Incorrect), a run of correct predictions (Dice+Correct), or no runs of prediction outcomes (Dice only). Horizontal bars indicate the average baseline probability of predicting a changed-dice outcome for each group. Error bars represent ±1 standard error of the mean. * *p* < 0.05, ** *p* < 0.01, and *** *p* < 0.001 reflect Wilcoxon signed-rank test against baseline within each group or pairwise Chi-squared comparisons across groups.

Gambler’s fallacy responding, as defined by elevated proportions of changed-dice outcome predictions relative to baseline on the critical trial, occurred only when a run of even or odd dice-sum outcomes was accompanied by a run of incorrect predictions. This was the case in both the controlled external-outcome conditions, in which dice outcomes (even or odd) were explicitly manipulated and prediction outcomes incidentally varied depending on participants’ responses, and in the controlled prediction-outcome conditions, in which prediction outcomes (correct or incorrect) were explicitly manipulated and dice outcomes incidentally varied depending on participants’ responses. In the controlled external-outcome conditions, for both the even-run (Fig 2A) and odd-run (Fig 2B) conditions, the proportion of changed-dice-outcome predictions was elevated when a run of a dice outcome was accompanied by a run of incorrect predictions (Dice+Incorrect, left plots; *W* = 348, *r* = 0.659, *p*<0.002, for the even-run and *W* = 720, *r* = 0.878, *p*<0.000002, for the odd-run conditions), but was not elevated when a run of a dice outcome was accompanied by a run of correct predictions (Dice+Correct, middle plots; *W* = 17, *r* = 0.258, *p*>0.475, for the even-run and *W* = 7, *r* = 0.467, *p*>0.416 for the odd-run conditions) or by no runs of a prediction outcome (Dice only, right plots; *W* = 26, *r* = 0.080, *p*>0.735, for the even-run and *W* = 94, *r* = 0.313, *p*>0.183, for the odd-run conditions).

**Fig 3:**
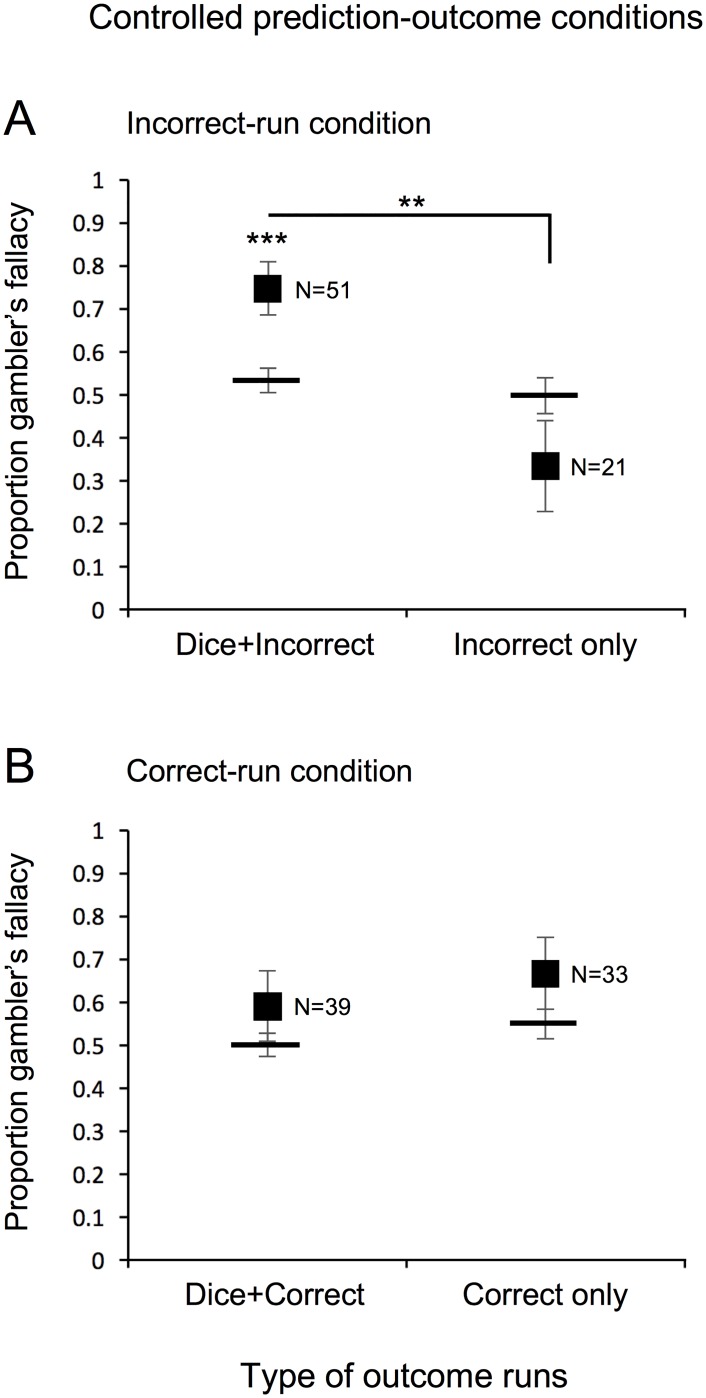
Experiment 1: Controlled prediction-outcome conditions. Proportion of participants predicting a changed-dice outcome (gambler’s fallacy) on the critical trial in the controlled prediction-outcome conditions following a run of (A) incorrect or (B) correct outcomes. Participants are grouped according to whether they concurrently had a run of a dice outcome (Dice+Incorrect for A or Dice+Correct for B) or not (Incorrect only for A or Correct only for B). Horizontal bars indicate the average baseline probability of predicting a changed-dice outcome for each group. Error bars represent ±1 standard error of the mean. * *p* < 0.05, ** *p* < 0.01, and *** *p* < 0.001 reflect Wilcoxon signed-rank test against baseline within each group or pairwise Chi-squared comparisons across groups.

The same pattern of results obtained for the controlled prediction-outcome conditions. In the incorrect-run condition (Fig 3A), the proportion of changed-dice-outcome predictions was elevated when a run of incorrect predictions was accompanied by a run of a dice outcome (Dice+Incorrect, left plot; *W* = 678, *r* = 0.511, *p*<0.002), but was not elevated when only a run of incorrect predictions occurred (Incorrect only, right plot; *W* = 57, *r* = 0.271, *p*>0.294). The proportion of changed-dice-outcome predictions was not elevated in the correct-run condition (Fig 3B) whether a run of correct predictions was accompanied by a run of a dice outcome (Dice+Correct, left plot; *W* = 210, *r* = 0.269, *p*>0.143) or only a run of correct predictions occurred (Correct only, right plot; *W* = 148, *r* = 0.264, *p*>0.188).

In addition, chi-squared tests indicated that participants who experienced concurrent dice and incorrect-prediction runs showed greater gambler’s fallacy responding on the critical trial than those who experienced any other type of run in the same condition (for even-run condition [Fig 2A], *χ*^2^[1] = 16.824, *p*<0.00005, for Dice+Incorrect run vs. Dice+Correct run, and *χ*^2^[1] = 24.289, *p*<0.000001, for Dice+Incorrect run vs. Dice-run only; for the odd-run condition [Fig 2B], *χ*^2^[1] = 6.688, *p*<0.01, for Dice+Incorrect run vs. Dice+Correct run, and *χ*^2^[1] = 8.500, *p*<0.004, for Dice+Incorrect run vs. Dice-run only; for the incorrect-run condition [Fig 3A], *χ*^2^[1] = 10.761, *p*<0.002, for Dice+Incorrect run vs. Incorrect-run only).

Thus, the results consistently suggest that gambler’s fallacy responding occurs only when a run of external outcomes is accompanied by a run of incorrect-prediction outcomes, over and above any baseline tendencies people may have to predict a changed external outcome. In particular, the results were the same whether attention was guided towards external outcomes in the even- and odd-run conditions (Fig 2) or attention was guided towards prediction outcomes in the correct- and incorrect-run conditions (Fig 3). This suggests that regardless of whether attention is guided toward external or prediction outcomes, a run of a dice outcome produces gambler’s fallacy responding only when it is concurrently accompanied by a run of failed prediction outcomes.

## Experiment 2 Materials and Methods

Methods are described only where they differ from experiment 1.

### Rationale

We conducted a second experiment to attempt to replicate the results from experiment 1 for the prediction-outcome conditions. We also verified that participants felt the sequences were truly random.

### Participants

One hundred participants recruited via Mechanical Turk (40 female; ages 18–70) gave their online informed consent to participate in the study following the procedures approved by Northwestern University's Institutional Review Board. None of these participants used the same Mechanical Turk ID numbers as those who participated in experiment 1.

### Procedure

Each participant performed the correct- and incorrect-run conditions in an order counterbalanced across participants. To determine whether participants felt both conditions represented random sequences, at the end of the experiment we asked, “Did you feel the outcomes of the trials [in the first/second sequence] were completely random? Yes/No.”

### Data validation

Using the same data validation methods we used in experiment 1, four participants who did not correctly respond to the questions following the presentation of the dice pictures in any run, and thirteen participants who guessed the same outcome (even or odd) on every one of the first 12 trials in all conditions, were eliminated from the analysis. Thus, analyses presented in the Results section include data from 83 participants.

## Experiment 2 Results

**Fig 4:**
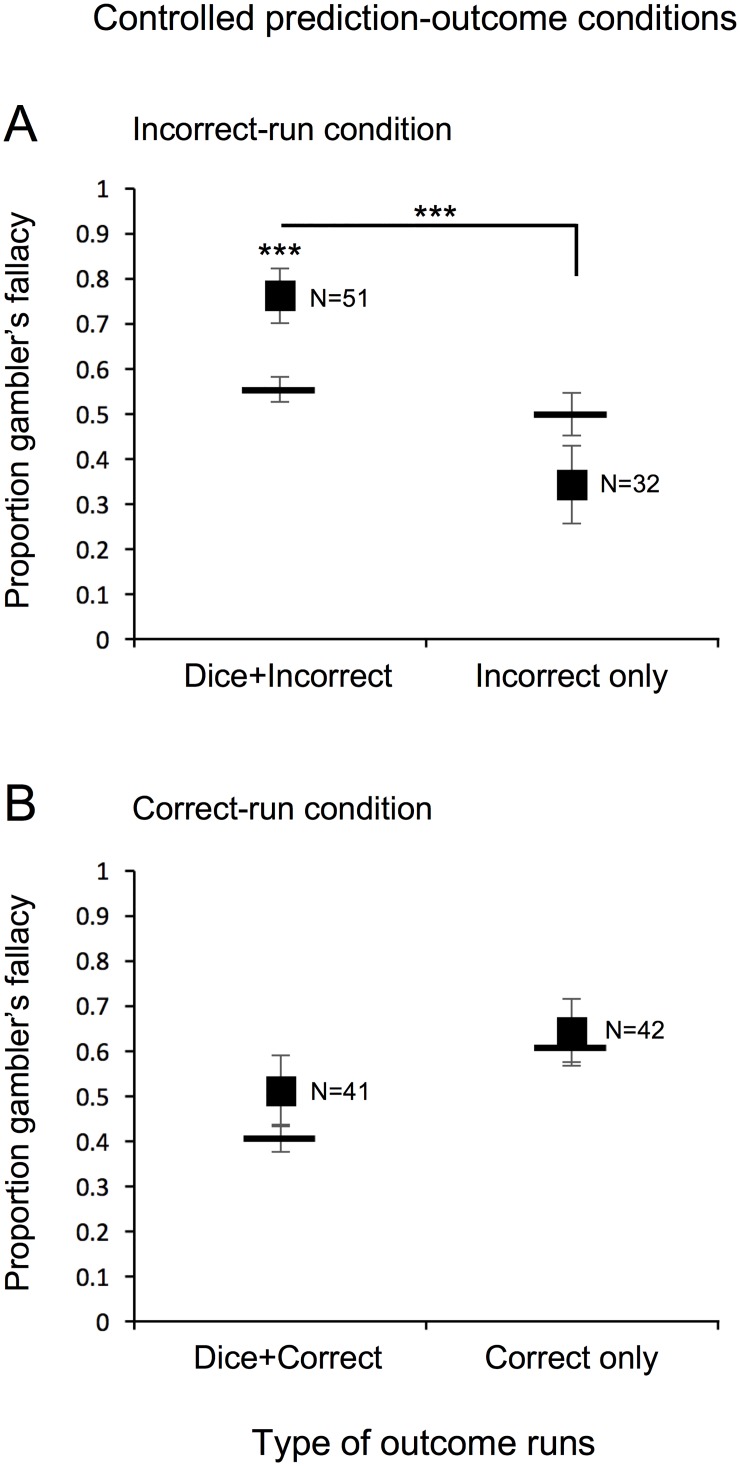
Experiment 2: Controlled prediction-outcome conditions. Proportion of participants predicting a changed-dice outcome (gambler’s fallacy) on the critical trial in the controlled prediction-outcome conditions following a run of (A) incorrect or (B) correct outcomes. Participants are grouped according to whether they concurrently had a run of a dice outcome (Dice+Incorrect for A or Dice+Correct for B) or not (Incorrect only for A or Correct only for B). Horizontal bars indicate the average baseline probability of predicting a changed-dice outcome for each group. Error bars represent ±1 standard error of the mean. * *p* < 0.05, ** *p* < 0.01, and *** *p* < 0.001 reflect Wilcoxon signed-rank test against baseline within each group or pairwise Chi-squared comparisons across groups.

We replicated the finding that gambler’s fallacy responding occurs only when a run of dice outcomes is accompanied by a concurrent run of incorrect predictions. In the incorrect-run condition (Fig 4A), the proportion of changed-dice-outcome predictions was elevated when a run of incorrect predictions was accompanied by a run of a dice outcome (Dice+Incorrect, left plot; *W* = 674, *r* = 0.550, *p*<0.0008), but was not elevated when only a run of incorrect predictions occurred (Incorrect only, right plot; *W* = 139, *r* = 0.320, *p*>0.134); this difference was significant (*χ*^2^[1] = 14.547, *p*<0.001). The proportion of changed-dice-outcome predictions was not elevated in the correct-run condition (Fig 4B) whether a run of correct predictions was accompanied by a run of a dice outcome (Dice+Correct, left plot; *W* = 252, *r* = 0.323, *p*>0.079) or only a run of correct predictions occurred (Correct only, right plot; *W* = 77, *r* = 0.089, *p*>0.621).

Most participants felt the outcomes of the trials in both conditions were completely random; the proportion of participants who felt outcomes were random was 78% for the first sequence and 70% for the second sequence (averaged across conditions) and 72% for the incorrect-run condition and 76% for the correct-run condition (averaged across order). There were no significant differences in the number of participants who felt the sequences were random between either the first versus second sequence or between the correct- and incorrect-run conditions (first vs. second sequence: *χ*^2^ = 1.54; correct-run vs. incorrect-run condition: *χ*^2^ = 0.495).

We thus replicated our primary finding, at least in the controlled prediction-outcome focus conditions, that gambler’s fallacy responding is driven by a combination of a run of external outcomes and a concurrent run of incorrect-prediction outcomes, even among participants who largely felt the sequences were random.

## Experiment 3 Materials and Methods

Methods are described only where they differ from experiment 2.

### Rationale

Experiment 2 replicated our findings in the controlled prediction-outcome conditions. In experiment 3, we attempted to replicate them in the controlled dice-outcome conditions. Here we administered the odd- and even-run conditions to separate groups of participants to determine whether gambler’s fallacy responding is driven by a run of dice outcomes accompanied by a concurrent run of incorrect predictions even when participants have no prior experience with the task. As in experiment 2, we asked the participants whether they felt the dice outcomes were completely random.

### Participants

One hundred ninety-eight participants recruited via Mechanical Turk (85 female; ages 18–70) gave their online informed consent to participate in the study following the procedures approved by Northwestern University's Institutional Review Board. One hundred participants performed the even-run condition and ninety-eight participants performed the odd-run condition. None of these participants used the same Mechanical Turk ID numbers as those who participated in either experiments 1 or 2.

### Procedure

Participants performed either the even- or the odd-run condition.

### Data validation

As in experiments 1 and 2, participants who did not correctly respond to the questions following the presentation of the dice pictures in the four trials of the run were removed from the analysis; four such participants were removed from the even-run condition and nine such participants were removed from the odd-run condition. Participants who guessed the same outcome (even or odd) on every one of the trials prior to the run were also eliminated from the analysis, resulting in the removal of ten participants from the even-run condition and five from the odd-run condition. Thus, analyses presented in the Results section include data from 86 participants in the even-run condition and 84 participants in the odd-run condition.

## Experiment 3 Results

**Fig 5:**
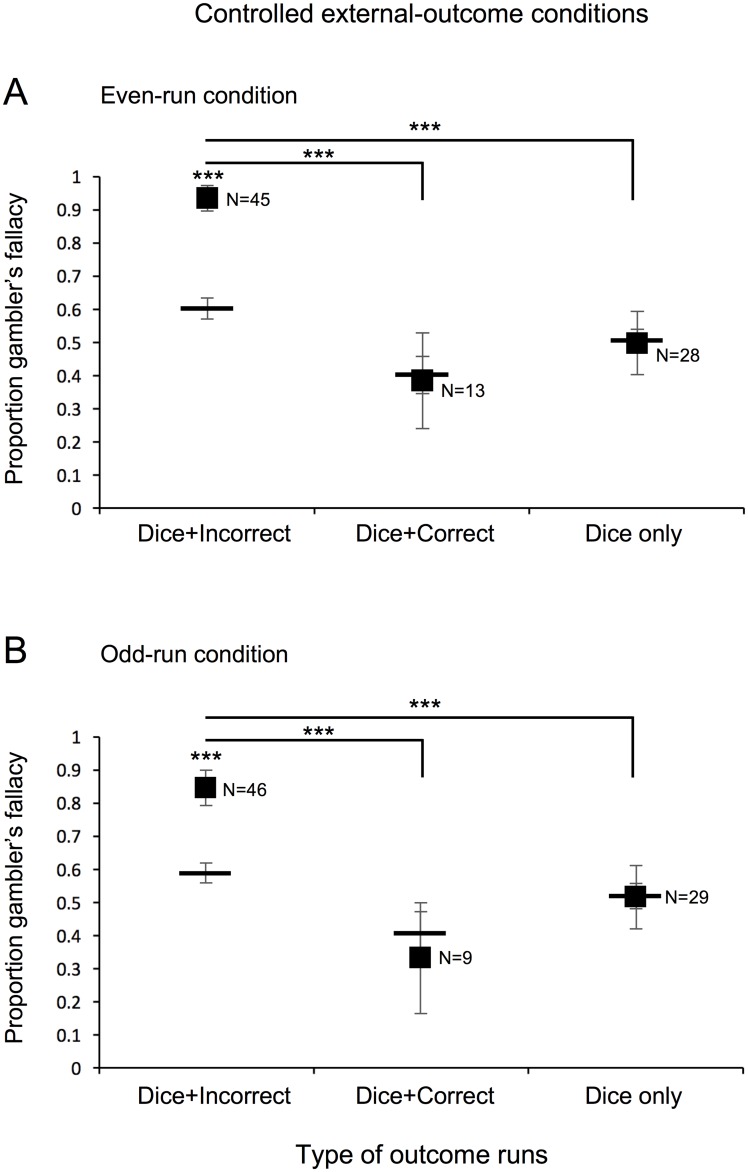
Experiment 3: Controlled external-outcome conditions. Proportion of participants predicting a changed-dice outcome (gambler’s fallacy) on the critical trial in the controlled external-outcome conditions following a run of (A) even or (B) odd dice outcomes. Participants were tested in either the even-run or odd-run condition whereas those in experiment 1 were tested in both conditions. Participants are grouped according to whether they concurrently had a run of incorrect predictions (Dice+Incorrect), a run of correct predictions (Dice+Correct), or no runs of prediction outcomes (Dice only). Horizontal bars indicate the average baseline probability of predicting a changed-dice outcome for each group. Error bars represent ±1 standard error of the mean. * *p* < 0.05, ** *p* < 0.01, and *** *p* < 0.001 reflect Wilcoxon signed-rank test against baseline within each group or pairwise Chi-squared comparisons across groups.

We again replicated the finding that gambler’s fallacy responding occurs only when a run of dice outcomes is accompanied by a concurrent run of incorrect predictions. For both the even-run (Fig 5A) and odd-run (Fig 5B) conditions, the proportion of changed-dice-outcome predictions was elevated when a run of a dice outcome was accompanied by a run of incorrect predictions (Dice+Incorrect, left plots; *W* = 756, *r* = 0.837, *p*<2.2x10^-6^, for the even-run and *W* = 614, *r* = 0.620, *p*<0.0004, for the odd-run conditions), but was not elevated when a run of a dice outcome was accompanied by a run of correct predictions (Dice+Correct, middle plots; *W* = 3, *r* = 0.033, *p*>0.943, for the even-run and *W* = 0, *r* = 0, *p* = 1, for the odd-run conditions) or by no runs of a prediction outcome (Dice only, right plots; *W* = 17, *r* = 0.042, *p*>0.854, for the even-run and *W* = 18, *r* = 0.041, *p*>0.853, for the odd-run conditions). Chi-squared tests indicated that participants who experienced concurrent dice and incorrect-prediction runs showed greater gambler’s fallacy responding on the critical trial than those who experienced any other type of run in the same condition (for even-run condition [Fig 5A], *χ*^2^[1] = 19.760, *p*<0.00001, for Dice+Incorrect run vs. Dice+Correct run, and *χ*^2^[1] = 18.143, *p*<0.00003, for Dice+Incorrect run vs. Dice-run only; for the odd-run condition [Fig 5B], *χ*^2^[1] = 11.039, *p*<0.001, for Dice+Incorrect run vs. Dice+Correct run, and *χ*^2^[1] = 24.079, *p*<0.000001, for Dice+Incorrect run vs. Dice-run only).

As in experiment 2, it appears that most participants felt the outcomes of the trials in both conditions were completely random (even-run condition: 82%; odd-run condition: 70%). There were no significant differences in the number of participants who felt the sequences were random between the even- and odd-run conditions (χ^2^[1] = 2.89). Further, focusing attention on the dice outcomes in this experiment did not influence the number of participants perceiving the trial outcomes as random relative to when participants were focused on the prediction outcomes in experiment 2 (experiment 3 [in which each participant received only one sequence, even-run or odd-run] vs. the first sequence given in experiment 2 [in which each participant received both the incorrect-run and correct-run sequences]; χ^2^[1] = 0.184).

Results from this experiment thus replicated the finding that gambler’s fallacy responding is driven by a combination of a run of external outcomes and a concurrent run of incorrect-prediction outcomes, even when participants had no prior experience with the task and mostly believed the outcomes were random.

## Experiment 4 Materials and Methods

### Rationale

Experiment 3 replicated our findings in the controlled external-outcome conditions. Based on the results of experiments 1–3 it appears that gambler’s fallacy responding may be primarily driven by experiencing a series of concurrent external outcomes and failed guesses. One concern is that these results might be specific to an online protocol, though there appears to be nothing peculiar about online dice outcomes that would make them unrepresentative of random outcomes in general. However, it is possible that people may behave differently when random outcomes are demonstrated in front of them. To address the issue of generalizability of the current results we performed a re-analysis of previous data [9] that one of us had obtained from a different prediction task in which participants predicted the outcomes of coin tosses that the experimenter performed in front of them. As the critical trial was the 8th trial in this experiment whereas it was the 16th trial in the current experiments 1–3, the re-analysis also helped to generalize the current results to different serial positions in a random sequence. Details of methods can be found in that paper; an overview is given here.

### Participants

One hundred twenty-four participants gave their written informed consent to participate in the study following the procedures approved by the Institutional Review Board at the University of Guelph. Four were dropped who reported not believing the announced outcomes were true on a post-study question, leaving 120 participants.

### Procedure

An experimenter stood in front of a room of participants, flipping a coin and calling out “heads” or “tails” in a pre-arranged sequence which included a run of four external outcomes as the 4^th^ through 7^th^ trials. Participants were led to believe that the experimenter truthfully announced each coin-toss outcome. Before each coin flip, participants recorded their prediction of the outcome of the flip and ranked their confidence in their prediction. After each outcome was announced, participants also recorded the actual outcome. The critical trial was the eighth trial, which followed four heads or four tails (counterbalanced).

### Data Validation

In our re-analysis, we removed data from 28 participants who incorrectly wrote down the actual “heads” or “tails” outcome after the experimenter called it out, as we assumed their attention was not focused on the task. Thus, the results were calculated from the prediction responses of the 92 remaining participants.

## Experiment 4 Results

The participants in this in-person coin-flip paradigm showed the same effect as we found with the online dice-sum paradigm used in experiments 1–3. Specifically, 46 of these participants experienced runs of incorrect predictions on two or more trials concurrent with the controlled external (heads or tails) runs; of these participants, 38 predicted the next trial in the direction of the gambler’s fallacy (*p*<4.7x10^-6^ in a binomial test against the chance level of 50%). Meanwhile, of the 10 participants who experienced runs of correct predictions on two or more trials concurrent with the controlled external runs, 4 predicted the next trial in the direction of the gambler’s fallacy (*p*>0.82). Finally, the remaining 36 participants experienced only the controlled external runs, and 22 of these predicted the next trial in the direction of the gambler’s fallacy (*p*>0.121). Thus, it again appears that only those participants who experienced runs of failed predictions concurrent with external-outcome runs made predictions on the critical trial that were consistent with the gambler’s fallacy.

## Overall Results and Discussion

Our results extend previous work on prediction behavior suggesting that gambler’s fallacy responses to runs of repeated external outcomes are particularly prevalent when prediction failures are experienced concurrently [5]. Experiments 1–3 consistently revealed that, regardless of whether people attend to the external outcomes (even vs. odd dice sums) or to the outcomes of their own predictions (correct vs. incorrect), people make gambler’s fallacy responses if and only if a run of repeated external outcomes is accompanied by a concurrent run of incorrect predictions (the Dice+Incorrect column in Table 1; see raw data and statistical tests in S1 –S6 Files). In none of the other conditions were gambler’s fallacy responses statistically significant, suggesting that concurrent runs of repeated external outcomes and prediction failures are both necessary and sufficient for gambler’s fallacy responding to occur. Although experiments 1–3 were conducted online, the re-analysis of existing data provided in experiment 4 confirms the results while random outcomes were physically demonstrated in front of participants and when the “critical trial” appeared in a different place in the sequence (see raw data in S7 File). Even if null results were to be considered inconclusive, at the very least, these results suggest that concurrent runs of prediction failures and external outcomes substantially strengthen gambler’s fallacy behavior.

The current results add context to the similar findings in Boynton’s research [5]. The results of those studies indicated that gambler’s fallacy responding increased following a prediction failure during a run of repeated external outcomes, but the history of prediction outcomes was not examined. In experiments 1–3 of the present study, the number of participants who experienced runs of repeated dice outcomes concurrent with runs of correct responses were substantially lower than the number of participants who experienced runs of repeated dice outcomes concurrent with runs of incorrect responses (Table 1). This suggests that people were more likely to continue making the same choice when they were losing than they were when they were winning. It is plausible, then, that in Boynton’s studies a disproportionate number of those participants in the failure condition who also experienced outcome runs would have experienced multiple prediction failures, although these were left unexamined. Therefore, it is possible that these multiple prediction failures largely account for the stronger gambler’s fallacy trend in Boynton’s data.

Interestingly, the current results may help explain a previous demonstration that gambler’s fallacy responses occurred when outcomes were presented one at a time, but were minimal when participants were shown a list of past outcomes (e.g., HTTHTHHH) and asked to predict a subsequent outcome [12]. Our results would predict minimal gambler’s fallacy responses in the list condition because people would not have the crucial experience of failed predictions while inspecting the list of past outcomes.

Why should runs of external outcomes interact with runs of prediction failures to produce gambler’s fallacy responding? One intuitive explanation that does not work well given the present results is the idea that gambler’s fallacy occurs because individuals inappropriately apply what they know about the statistics of longer sequences of events (e.g., 50% even or odd) to shorter sequences of events [13]. The problem with this explanation is that if it were true, individuals who experienced any type of run of dice outcomes should have produced gambler’s fallacy responding in experiments 1–3 regardless of prediction outcomes; clearly, this is not the case. A competing explanation that is more likely to be useful in light of the present results emphasizes the tendency to view similar independent events occurring in a sequence as somehow linked [14]. This linkage of similar events across time could support the idea that individuals perceive even random events as a system that is possible to engage. For example, in a recent study, gambler’s fallacy responding occurred after a tails-heads-heads-heads sequence, but not after a heads-heads-heads-tails sequence, even though both runs contained the same number of heads and tails outcomes, suggesting that it is not the recent frequency of outcomes but their order that is motivating gambler’s fallacy responding, in an attempt to find closure to a sequence [15]. However, this gestalt explanation does not explain why closure is sought only after a run of prediction failures, as shown in the current results.

To bridge this gap, we could extend the gestalt explanation to take the influence of previous prediction errors into account. Although speculative at this point, we propose an “error-driven gestalt” explanation, suggesting that prediction failures draw attention to the lack of fit between the person and the system he/she is attempting to predict (in this case, picking dice outcomes), and highlighting this mismatch leads the person to attempt to seek closure. It is not a new idea that decision behavior is driven by attempts to gain a sort of mastery over a system—it was proposed by Langer [16], who built on the work of DeCharms [17]. Here, we speculate that attempts to find closure are triggered by a consistent mismatch between what an individual expects and what occurs (i.e., driven by consistent error). When prediction failures occur as a result of outcomes that have no consistent pattern, there is no obvious solution to the problem, at least in a system presumed to be random. This may explain our finding that with prediction failures alone, without a simultaneous external-outcome run, there is no consistent response pattern. However, when there is a run of external outcomes concomitant with a run of prediction failures, people may engage in gambler’s fallacy responding in an attempt to gain closure (i.e., to stop the failures). For example, in the paradigm used for experiments 1–3, choosing “odd” three times in a row, but having the outcome come up as “even” all three times likely gets the person’s attention. Assuming that individuals believe the outcomes are random, this pattern may lead them to stay with the same prediction, knowing that the run will have to end eventually. In contrast, following correct predictions, we propose that there is no error-driven attempt to find closure because an experience of the predicted outcome provides a cognitive closure. In a sense, a win may completely “reset” error-driven attempts to find closure until the next loss, producing no predictable response behavior after a win. Research aimed at testing this explanation could examine differences in strategies that people employ during runs of successes versus failures, while taking into account the role of external outcomes and desire for closure in shaping those strategies.

## Conclusions

We have demonstrated that gambler’s fallacy behavior, or predicting that a different external outcome becomes more likely following a run of the same external outcomes, nearly completely depends on concurrent runs of prediction failures. Thus, our understanding of the gambler’s fallacy in particular, and perhaps predictive behavior in general, may benefit from simultaneously taking into account both the outcomes of the system that is being predicted (external outcomes) and the outcomes of the predictions people make regarding that system (prediction outcomes). In general, it is our conviction that future experiments that examine these external and internal factors together will bring us a long way towards discovering an effective theory of predictive behavior.

## Supporting Information

S1 File Experiment 1 Raw and Mean Data in Excel format. (XLSX)

S2 File Experiment 1 Wilcoxon tests versus baseline in Excel format. (XLSX)

S3 File Experiment 2 Raw and Mean Data in Excel format. (XLSX)

S4 File Experiment 2 Wilcoxon tests versus baseline in Excel format. (XLSX)

S5 File Experiment 3 Raw and Mean Data in Excel format. (XLSX)

S6 File Experiment 3 Wilcoxon tests versus baseline in Excel format. (XLSX)

S7 File Experiment 4 Raw and Mean Data in Excel format. (XLSX)
